# Potential Use of Tannin Extracts as Additives in Semen Destined for Cryopreservation: A Review

**DOI:** 10.3390/ani12091130

**Published:** 2022-04-28

**Authors:** Mohammed S. Liman, Abubeker Hassen, Lyndy J. McGaw, Peter Sutovsky, Dietmar E. Holm

**Affiliations:** 1 Department of Production Animal Studies, Faculty of Veterinary Science, University of Pretoria, Pretoria 0110, South Africa; limanms69@gmail.com; 2 Niger State Livestock and Fisheries Institute, Ministry of Livestock and Fisheries Development, Minna 920001, Niger State, Nigeria; 3 Department of Animal and Wildlife Sciences, Faculty of Natural and Agricultural Science, University of Pretoria, Pretoria 0028, South Africa; abubeker.hassen@up.ac.za; 4 Phytomedicine Programme, Department of Paraclinical Sciences, Faculty of Veterinary Science, University of Pretoria, Pretoria 0110, South Africa; lyndy.mcgaw@up.ac.za; 5 Division of Animal Sciences, Department of Obstetrics, Gynecology and Women’s Health, University of Missouri, Columbia, MO 65211-5300, USA; sutovskyp@missouri.edu

**Keywords:** cryopreservation, spermatozoa, tannin, polyphenols, semen additives, antioxidant

## Abstract

**Simple Summary:**

Freezing of semen used for artificial reproductive technologies (ART) affects the survival and vigour of sperm cells due to excessive production of reactive oxygen species (ROS) during the freezing and thawing processes. ROS plays a physiological role in sperm function but excessive ROS production from damaged sperm cells can hinder sperm’s motility and their ability to fertilise an oocyte. Tannins, a class of water-soluble plant polyphenols, are known to have antioxidant and other health-promoting effects and may serve as binders/acceptors to reduce the deleterious effects of excessive ROS produced during the freezing and thawing process. This review is the first to analyse the available data supporting the use of tannins as additives to semen extenders to improve the survival of cryopreserved spermatozoa during storage and after thawing. It is concluded that tannins and their derivatives have naturally protective properties with the potential to ameliorate sperm cell survival after freezing.

**Abstract:**

Cryopreservation and storage of semen for artificial insemination (AI) result in excessive accumulation of reactive oxygen species (ROS). This leads to a shortened life span and reduced motility of spermatozoa post-thawing, with consequent impairment of their function. However, certain levels of ROS are essential to facilitate the capacitation of spermatozoa required for successful fertilisation. Tannins, as well-known antioxidant compounds, may act as ROS binders/acceptors/scavengers to inhibit the damaging effects of ROS. This review comprises an analysis of the semen cryopreservation protocol and health functions of tannins, as well as the effects of ROS on fresh and cryopreserved semen’s longevity and fertilisation. Additionally, we surveyed available evidence of the effects of tannin extract feed supplementation on male fertility. We furthermore interrogated existing theories on tannin use as a potential additive to semen extenders, its relationship with semen quality, and to what degree existing theories have been investigated to develop testable new hypotheses. Emphasis was placed on the effects of tannins on ROS, their involvement in regulating sperm structure and function during cryopreservation, and on post-thaw sperm motility, capacitation, and fertilising ability. The diverse effects of tannins on the reproductive system as a result of their potential metal ion chelation, protein precipitation, and biological antioxidant abilities have been identified. The current data are the first to support the further investigation of the incorporation of tannin-rich plant extracts into semen extenders to enhance the post-thaw survival, motility, and fertilising ability of cryopreserved spermatozoa.

## 1. Introduction

Cryopreservation reduces the functional and structural integrity of spermatozoa due to the development of reactive oxygen species (ROS) [1,2]. ROS are produced during numerous chemical reactions in different parts of the mammalian body [1]. In the testes, ROS are produced during spermatogenesis within the seminiferous tubules and steroidogenesis in the interstitium [3]. Cryopreservation and storage of semen lead to changes in the sperm mitochondrial membrane and the resident electron transport chain [3], which result in the excessive release of ROS, hydrogen peroxide (H_2_O_2_), nitric oxide (NO), or superoxide anion (O_2_^−^), with consequences on sperm capacitation and the acrosome reaction [2]. Cryoprotectants are important for the cryo-survival of spermatozoa [4], and these may include egg yolk, glycerol [4], dimethyl sulphoxide (DMSO) [5], ethylene glycol [6], Triladyl^®^ (a commercially available semen extender) [7], and butylated hydroxytoluene (BHT) [8,9,10,11]. Combinations of cryoprotectants such as glycerol and ethylene glycol [7,12] and acetamide together with lactamide [13] may also be employed. Antioxidant substances may reduce the impact of oxidative stress and thereby improve the quality of semen post-thawing [14]. Cryoprotectants are important for the cryo-survival of spermatozoa [15].

Low levels of ROS are, however, associated with increased sperm motility, viability, increased capacity for successful fertilisation during sperm–oocyte interactions, and fertility in mammalian species [16]. Antioxidant additives in semen diluents for cryopreservation should therefore not aim to eliminate ROS [17]. When ROS occur in small concentrations, they act as mediators of normal sperm function, whereas when present in excess, they are toxic to spermatozoa [14]. 

Sperm capacitation normally occurs in the oviduct and involves biochemical and structural changes that make the spermatozoa competent to attach to the zona pellucida of the oocyte, penetrate it, and fuse with the oolemma [18]. The cellular changes that occur include the activation of soluble adenyl cyclase that produces cAMP, the influx of Ca^2+^ ions, Zn ions [19,20], efflux of cholesterol from the plasma membrane, leading to its fluidity/fuseability, and the generation of more ROS, with a consequent increase in intracellular pH [7]. Additionally, activation of protein kinase A and downstream protein tyrosine kinases results in the protein phosphorylation of numerous proteins on tyrosine residues [21]. This process results in the hyperactivation of sperm tail motility, which is necessary for sperm detachment from the oviductal sperm reservoir and the penetration of the egg vestment at fertilisation. It was reported that controlled and low ROS generation plays a physiological role during the capacitation and acquisition of sperm’s fertilising ability, with ROS-specific scavengers inhibiting the process [14,22,23]. These processes of ROS affecting the spermatozoa have been reviewed previously [24].

Thus, ROS homeostasis appears to be equally important for timely sperm capacitation within the female oviduct, and for the prevention of premature capacitation during semen processing and cryopreservation for artificial insemination (AI).

Plants contain combinations of complex polymeric phenols, which are amongst the most studied phytochemicals because of their diverse array of useful biological functions and health-promoting effects [14]. Consequently, their antioxidation effects on the production of ROS, sperm longevity, and fertilising potential were reviewed using the available peer-reviewed data on tannin extract supplementation for male fertility. The aim was to document the utilisation of the biological and reproductive health benefits of tannins, with a view to exploiting their potential for use as additives to improve the cryopreservation of semen. To our knowledge, this review is the first to recommend further structured evaluation of the value of tannin extracts or compounds as additives into semen destined for cryopreservation [14,25].

## 2. Methodology

This theoretical literature review (TLR) focused firstly on the existing evidence of the biological and health benefits of tannins, specifically with regard to their antioxidant properties and resultant inhibitory effects on lipid peroxidation, as well as their antiviral, antibacterial, and anti-inflammatory effects in terms of protecting spermatozoa against microbial infections during semen processing, cryopreservation, and distribution. This first section is divided into three subsections addressing the cryopreservation of semen using tannins, and their relevant biological and health functions, respectively. Secondly, we investigated the current evidence on the effect of ROS on sperm viability/semen longevity, and on the requirement for low levels of ROS in semen fertility. 

**Figure 1:**
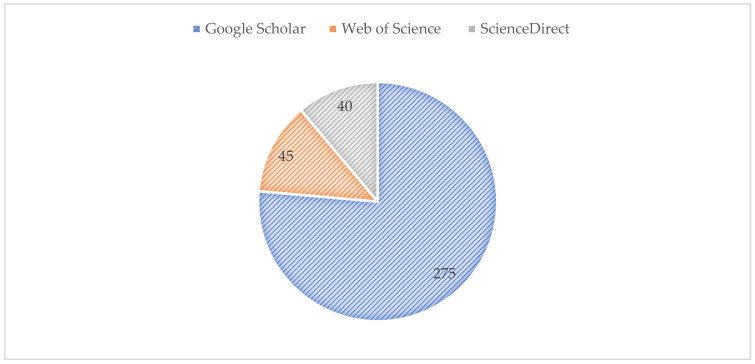
Database of the number of reports from Google Scholar, Scopus, and Web of Science on tannin additives for cryopreservation (*n* = 844).

Articles used in this review had a concise hypothesis, with keywords searched on databases including Google Scholar, Scopus/ScienceDirect, and Web of Science/Pubmed (which includes CABAbstracts, Medline and Zoological Records). Inclusion words: “additives”; “cryopreservation”, “tannin-extracts” or “fractions”, or “compound” and “health” or “biological” with emphasis on specific functions, namely “antiviral”, “antibiotic”, “antioxidant”, and “protection against lipid peroxidation”, excluding anticancer and antidiabetes. Database results for Google Scholar (645, 76%); Scopus/ScienceDirect (94, 11%); and Web of Science/Pubmed (105, 13%) are all published reports, respectively (Figure 1). The relevant reports used are represented as cited in this study. 

## 3. Effect of ROS on Cryopreserved Spermatozoa

### 3.1. ROS Effect on Sperm Cryopreservation and Longevity 

**Figure 2:**
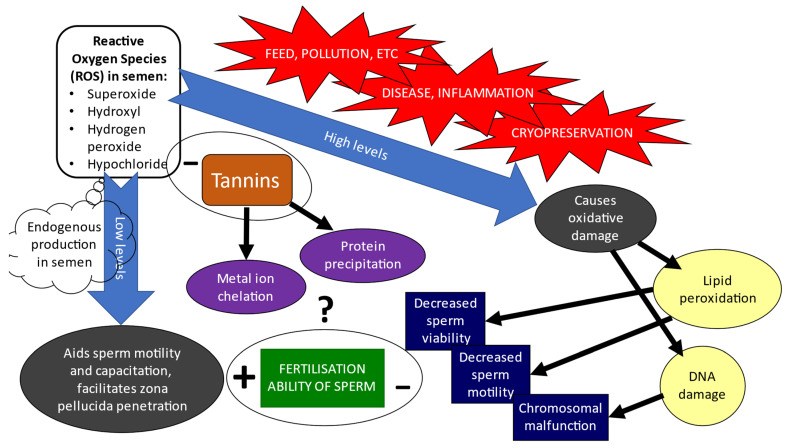
Demonstration of the homeostatic effects of ROS on sperm’s fertilisation ability, and the potential benefits of tannins to ameliorate its effects, particularly following cryopreservation.

ROS are a group of molecules (free radicals, oxygen ions, peroxides, etc.) that are produced during aerobic metabolism in the mitochondria of cells, and are important components of physiological processes and cellular signalling events [1]. The liquid or frozen semen preservation and its effect on semen quality were reviewed previously [14]. Oxidative damage in semen impairs spermatozoal function, resulting in a loss of motility, loss of mitochondrial activity, increase in deoxyribonucleic acid (DNA) damage, and lack of activation of apoptotic pathways [26]. Consequently, unresolved issues affecting fertility are encountered in artificially collected semen samples, such as infections, inadequate constituents of semen extenders and protocols adopted during cryopreservation processes, and the overall need for highly skilled intra-uterine insemination. Mammalian spermatozoa naturally contain antioxidants and ROS scavenging enzymes, such as glutathione (GSH), superoxide dismutase, and catalase (CAT) [27]. These endogenous antioxidants often are not sufficient to prevent lipid peroxidation during cryopreservation [28]. Excess ROS that develop during the storage of spermatozoa are largely responsible for damage to spermatozoa. The damage of the sperm plasma membrane due to the effect of ROS consequently exposes semen to lipid peroxidation, resulting from the high content of polyunsaturated acids, and DNA damage [29]. Thus, the cryopreservation of semen is dependent on the reversible reduction of the survival and metabolic activity of spermatozoa [30]. This could be achieved by the provision of an effective environment for the steady cooling of semen, with a focus on the development of extenders that maintain membrane integrity, increase motility, maximise sperm’s ability to capacitate, prevent oxidative stress, and minimise the generation of reactive oxygen species (ROS) during cryopreservation and storage [31,32,33]; see Figure 2.

The cytotoxic action of ROS on spermatozoa is mediated by high concentrations of phospholipid-bound polyunsaturated fatty acids (PUFA) in the sperm plasma membrane, especially docosahexaenoic acid (DHA), with six double bonds per molecule, which makes them susceptible to free radical attack [34]. Additionally, spermatozoa lack an enzyme apurinic/apyrimidinic endonuclease (APEI), which plays a significant role in DNA repair and base excision repair pathways [24]. Furthermore, the sperm DNA is hyper-condensed; thus, it is not easily accessible to repair mechanisms.

### 3.2. Effects of ROS on Sperm’s Fertilising Potential

Low levels of ROS are associated with increased sperm motility, viability, increased capacity for fertilisation during sperm–oocyte fusion, and general male fertility in mammalian species [16]. When ROS are in low concentrations, they act as mediators of normal sperm function, whereas in excess, they are toxic to spermatozoa. Sperm capacitation is a complex process by which spermatozoa acquire the ability to fertilise the mature oocyte. This occurs within the oviductal sperm reservoir and involves the biochemical and morphological changes that make the spermatozoon competent to attach to the zona pellucida of the oocyte, penetrate it, and fuse with the oolemma [18]. Conception rates in livestock AI depend on the quality of semen, which is generally low post-thawing, with the capacitation and fertilisation processes being dependent on the effect of the sub-lethal dysfunction of spermatozoa [35]. Premature sperm capacitation brought about by cryopreservation and thawing is referred to as cryocapacitation [36] and, similarly to physiological capacitation, is irreversible and terminal, leading to a shortened sperm lifespan and eventual death before spermatozoa can reach the oviductal fertilisation site following AI. The selection of animals with good-quality semen for cryopreservation and AI is a critical step in improving the fertility levels of frozen–thawed semen [37,38]. Despite having satisfactory fertility testing in terms of fresh-stored semen, the frozen–thawed semen of some animal species does not meet standards of acceptable fertilisation results suitable for commercial AI programmes [38,39]. Accumulated evidence indicates that inherent male progeny variability is one of the factors in semen cryopreservation responsible for the marked differences in sperm cryo-survival [37,38,39,40]. Individual differences in sperm quality and cryo-survival are addressed by ongoing efforts to identify gene variants and differentially expressed sperm proteins associated with either high or low sperm cryotolerance in livestock species [41,42].

## 4. Tannins

### 4.1. Properties of Tannins

Tannins are sourced from a multitude of trees and shrubs. Notable for industrial importance are black wattle or black mimosa (Mimosa tannin, *Acacia mearnsii*), quebracho wood (*Schinopsis lorentzhii*), oak bark (*Quercus robur*), chestnut wood (*Castanea sativa*), mangrove wood (*Algarobilla chilena*), gambir (*Uncaria gambir*), the bark of several species of pines and firs, such as *Pinus radiata* and *Pinus nigra*, as well as many other plants harbouring extractable tannins [43,44,45,46]. Tannins are a renewable resource used in several fields, ranging from the traditional application of tanning to producing heavy-duty leather and as wood adhesives up until the 1960s and 1970s, whereafter new applications were investigated [44], such as the proposed use of chestnut tannin as an antimicrobial and to reduce mycotoxins [47]. Tannins dissolve in water to form colloidal solutions, with their solubility dependent on the degree of polymerisation [48]. They are soluble in alcohol and acetone, and react with ferric chloride [49]. They have moderate stability in aqueous solutions, especially during extraction with boiling water (decoctions), in which they decompose in 30 min into gallic acid, ellagic acid, and corilagin [44]. At the centre of hydrolysable tannins is a polyol carbohydrate (D-glucose), which is partially or completely esterified with a phenolic group such as gallic acid (gallotannins) or ellagic acid (ellagitannins). Hydrolysable tannins are hydrolysed by weak acids or weak bases to produce carbohydrates and phenolic acids. Condensed tannins (proanthocyanidins) are polymers of 2–50 (or more) flavonoid units joined by carbon-to-carbon bonds, which are not easily cleaved by hydrolysis.

### 4.2. Extraction of Tannins

Tannins, both hydrolysable and condensed, are commonly extracted with a mixture of water and acetone. Optimal yield may be obtained from fresh, frozen, or lyophilised material. Some tannin-rich extracts are available from varied sources and are used as supplements to improve reproduction.

### 4.3. Medicinal Properties and Biological Functions of Tannins

The health benefits of tannins include antioxidant, anti-carcinogenic, cardioprotective, antimutagenic, antiviral, antibacterial, haemostatic, and anti-inflammatory properties, as well as inhibition of lipid perioxidation [45,46] Hydrolysable tannins are often cited for their antimicrobial activity [46] and chemopreventive properties against degenerative diseases [50]. These multi-functional properties of tannins are utilised in the treatment of human diseases [51]. Hydrolysable tannins are also inhibitors of α-glucosidase, which is an enzyme known to be involved in the modulation of the absorption of glucose in tissues [48]. 

Antioxidants have been used in semen extenders, including cysteamine, taurine, trehalose, and selenium, to improve the motility, viability, and membrane integrity of post-thawed semen [52,53], with significant results. Some other antioxidants, such as Vitamin C and E and catalase, have been used to supplement human, cattle, boar, rabbit, and stallion semen [54,55] In a study of the α-glucosidase inhibition and antioxidant activity of an oenological commercial tannin (Tan’Activ^®^ toasted oak wood *Quercus robur*), the extraction and fractionation process yielded four fractions, with one of the fractions generating a sub-fraction with enhanced α-glucosidase inhibitory activity with an inhibitory concentration (IC_50_) of 6.15 µg/mL [56]. The oak wood is used for barrel staves in the winemaking process and the polyphenols are not only used in the ageing of wine but in maintaining aroma/flavour, as well as contributing useful health properties [57,58].

Synthetic water-soluble polymers such as polyvinyl pyrrolidone (PVP) and polyethylene glycol (PEG) are used as tannin-binding agents for quantification and to neutralise the negative effect of tannins in animal diets [49]. The PVP is used to bind hydrolysable tannins, while PEG is used for condensed tannins. These groups of tannins both contain sufficient oxygen molecules in their chains to form strong hydrogen bonds, with the phenolic and hydroxyl groups in tannins serving to precipitate them from solutions [49]. 

### 4.4. Use of Tannins as Supplements to Improve Reproduction Outomces or as Semen-Protective Agents

Tannin extracts or compounds are extracted using ethanol or water into powdered substances and stored at −20 °C [56,59,60] for later use as supplements (feed) (Table 1) or added into semen extenders (Table 2), etc., after optimisation. 

Certain tannin concentrations have exerted efficiency in fertilisation, but with no effect on sperm kinematic parameters, acrosome integrity, mitochondrial membrane integrity, lipid perioxidation, or capacitation status or its viability [73]. The ethanol extract of a commercial oenological tannin (*Quercus robur*, toasted oak wood Tan’Activ^®^) had a biological effect at a concentration of 10 µg/mL, stimulating an increase (*p* < 0.001) in in vitro swine sperm capacitation at the tail principal piece (B pattern) and increased (*p* < 0.001) oocyte fertilisation rate [60]. However, at 100 µg/mL, the opposite effect was recorded on both sperm capacitation (B pattern) and fertilising ability, associated with higher sperm viability [60]. Where 5% crude tannin was added to the semen of the Bali breed of cattle for 14 days, it increased (*p* > 0.001) motility and viability, with a decrease in abnormal semen [76]. Guava (*Psidium guajava*) leaf extract, comprising 3% crude tannin, was added to liquid semen (stored for 15 days at 4–5 °C) of Ettawa crossbred Boer goats and improved (*p* < 0.001) the motility and viability and maintained intact plasma membranes of the spermatozoa, while a concentration of 24% of the crude tannin reduced viable sperm content [71]. Altogether, it appears that tannins may benefit extended semen through ROS scavenging and microbial growth limitation. It is yet to be determined if tannins may also convey cryotolerance during semen preservation.

## 5. Conclusions

To our knowledge, this is the first review recommending the addition of tannin-rich extract or compounds into semen destined for cryopreservation, exploiting their diverse effects on biological systems due to their potential for metal ion chelation and biological antioxidation. The varied biological roles, however, together with the enormous structural variations of these compounds, make it difficult to develop a model that allows accurate prediction of the role of tannins in any biological system. Therefore, it becomes imperative for studies to be conducted on tannin biological activities by determining their chemical structure, biological activity, and structure–activity relationships so that potential applications can be explored. While the inquiry into the biological activities of tannins is still in its infancy, it holds a promise of utility in livestock-assisted reproductive technology and human reproductive therapy. The addition of plant tannin extracts, extract fractions, or purified/synthetic compounds derived therefrom to semen may elevate the quality and viability of semen intended for cryopreservation. Beyond sperm cryopreservation, protocols for semen collection, processing, and liquid semen distribution in relevant livestock species could benefit from judicious, experiment-validated tannin supplementation, taking advantage of the antioxidant properties of tannins.

## Figures and Tables

**Table 1:** Reported effects of tannin extracts used as food/feed supplements on reproduction outcomes in humans or animals.

Plant from Which Tannins Were Extracted (References)	Compounds Identified	Extraction Method	Subject (Animal Species and Gender)	Effect on Reproduction
*Zingiber officinale* (ginger) root extract [61]	High content of total flavonoids, tannins, alkaloids, and total phenolic components	Ethanol	*Rattus rattus* (Rat)—male	Restored testis histopathological alterations, reduced arsenic, and improved sperm parameters.
*Spondias mombin* leaf extract [62]	Leaves contain saponins, alkaloids, flavonoids, tannins, steroids, phenolics, phlobatannins, cardiac glycosides, cardenolides, and dienolides with saponins	Ethanol	*Cavia porcellus* (Guinea pig)—male	Induced infertility in males via endocrine dysregulation, anti-spermatogenic activity, testicular dysfunction, and antioxidative stress.
*Allium triquetrum* (wild garlic) bulb and leaf extract [63]	Tannins (leaves have higher concentration)	Water	*Rattus norvegicus* (Wistar rat)—male	Used in the treatment of reproductive toxicity of lead acetate by reducing lead testicular injury by boosting sperm characteristics and ameliorating oxidative sperm markers.
*Azadirachta indica* leaf and fruit extracts [64]	Not reported	Methanol	*Rattus norvegicus* (Long Evans rat)—male	At 200 µg/mL, increased percentage of morphological defects. (Cellular detachment in the seminiferous epithelium with sperm death without decrease in number of sperm).
*Acacia mearnsii* (Black Wattle) bark extract [65]	Condensed tannins average MW 1250 (500 to 3000), non-tannin polyphenols, salts, sugars, and organic acids. Total tannins (65.5%), tannic acid, and condensed tannin (30.5%) as leucocyanidin	Water	*Ovis aries* (Sheep: mutton merino)—male	Increase in testicular length, semen volume, semen concentration, and reduction in sperm with morphological defects.
*Phoenix dactylifera* (Date palm) fruit extract [66]	Review study		*Homo sapiens* (Man)—male and female	It has a potent effect on male hormones, seminal vesicle parameters, and sperm motility and viability.
*Turraea fischeri* bark extract [67]	20 compounds including several isomers of flavonolignan cinchonain-I and dominant bis-dihydrophenoxyl propanoid-substituted catechins hexsoides	Methanol	*Rattus norvegicus* (Wistar rat)—male	Enhanced reduction in the elevated levels of aspartate aminotransferase (AST), malondialdehyde (MDA), and increased glutathione (GSH) content in the liver.
*Mucuna pruriens* (Thai (T-MP)) seed extract [68]	Not reported	Water	*Rattus rattus* (Rat)—male and female	Exhibit antioxidation capacity, phytoestrogenic effect on females, and increased testicular and sperm markers of male fertility.
*Vitis vinifera* (Grape) seed tannin extract (GPE) [69]	GPE has a 95% purity coefficient (56.5% condensed tannins)	Not reported	*Ovis aries* (Hu lambs)	Improved the seminiferous tubules’ development, diameter, and increase in Sertoli cells. Also increase in superoxide dismutase (SOD).
*Caesalpinia pulcherrima* bark extracts [70]	Alkaloids, flavonoids, steroids, and triterpenes	Water and ethanol	*Rattus rattus* (Rat)—female	Reduced ovarian size and increased uterine weight.

MW = Molecular weight.

**Table 2:** Tannin-rich extracts used as semen-protective agents in humans and various domestic animals.

Plants from Which Tannins Were Extracted (References)	Compounds Identified	Extraction Methods	Subject (Animal Species and Gender)	Effect on Sperm
*Psidium guajava* (Crude guava) leaf tannin extract [71]	2.41% of tannin, 20.80% of phenols per 17.825 g of extract	Methanol, ethyl acetate, and acetone	*Capra aegagrus hircus* (Etawa crossbred goat)	At 3%, increase in sperm motility, viability, and maintained intact plasma membrane integrity.
*Aspalathus linearis* (Rooibos) extracts [72]	Major flavonoids, flavols, and low tannins	Water	*Sus scrofa domesticus* (Pig)	Enhanced the sperm velocity, protected acrosome integrity, and preserved membrane integrity during 96 h of storage.
Mixture of chestnut and Quebracho wood (60/40) tannin-rich vegetal extract [73]	94.2% tannin content	Filter Freiberg-hide powder method	*Sus scrofa domesticus* (Pig)	Increased penetration rate with oocytes inseminated with thawed sperm pretreated with vegetal extract, and at 5 µg/mL, it exerts total efficiency on fertilisation.
*Entada abyssinica* (Splinter bean) bark extract [74]	28 compounds including tannins and gallic acid derivatives	Methanol	*Ovis aries* (Sheep)	Increased post-thaw progressive sperm motility, plasma membrane integrity, % of intact sperm increased with decrease in apoptotic/necrotic sperm.
*Quercus robur* (Toasted oak wood) (Tan activ^®^) [56,60]	Monogalloyl glucose (332.2), Glucose esterified by hexahydroxydiphenic acid (482.2), Gallic acid (170.1), Ellagitannins, castalin (632.4), Vescalsgin (934.6), Grandinin or its isomer roburin E (1066.7)	Ethanol	*Sus scrofa domesticus* (Pig)	Stimulated the sperm capacitation and oocyte fertilisation rate in a swine in vitro fertilisation trial.
*Capparis spinosa* leaf extract [59]	Flavones and flavanols, total flavonoids, total phenolic content, tannins, and the total carbohydrates	Water and ethanol	*Homo sapiens* (Man)	Increased progressive, total in vitro motility, viability, and maintained sperm DNA integrity.
*Avena sativa* (Oats) seed extract [75]	Phenols—93.2 mg/g, Flavonoids—67 mg/g, Saponins—5.9%, Glycosides—17.6%, Terpenoids—4.6%, Rutin—179 ppm, Kaemperol—513 ppm, Quercetin 409 ppm, Gallic acid—348 ppm	Water	*Bos taurus* (Bovine: Holstein)	Improved sperm individual motility, viability, plasma membrane integrity, and acrosome integrity.

## Data Availability

The data presented in this study are available upon request from the corresponding author.
